# EMD in periodontal regenerative surgery modulates cytokine profiles: A randomised controlled clinical trial

**DOI:** 10.1038/srep23060

**Published:** 2016-03-15

**Authors:** Oscar Villa, Johan C. Wohlfahrt, Odd Carsten Koldsland, Steven J. Brookes, Staale P. Lyngstadaas, Anne M. Aass, Janne E. Reseland

**Affiliations:** 1 Department of Biomaterials, Institute of Clinical Dentistry, University of Oslo, Oslo, Norway; 2 Department of Periodontology, Institute of Clinical Dentistry, University of Oslo, Oslo, Norway; 3 Department of Oral Biology, Leeds Dental Institute, University of Leeds, Leeds, UK

## Abstract

The enamel matrix derivative (EMD) contains hundreds of peptides in different levels of proteolytic processing that may provide a range of biological effects of importance in wound healing. The aim of the present study was to compare the effect of EMD and its fractions on the cytokine profiles from human gingival fibroblasts *in vitro* and in gingival crevicular fluid (GCF) in a randomized controlled split-mouth clinical study (n = 12). Levels of cytokines in cell culture medium and in GCF were measured by Luminex over a 2-week period. In the clinical study, levels of pro-inflammatory cytokines and chemokines were increased, whereas the levels of transforming growth factor-α (TGF-α) and platelet-derived growth factor-BB (PDGF-BB) were reduced. The *in vitro* study showed that EMD and its high and low molecular weight fractions reduced the secretion of pro-inflammatory cytokines and chemokines compared to untreated cells. EMD had an effect on levels of cytokines related to fibroplasia, angiogenesis, inflammation and chemotaxis both *in vitro* and *in vivo*, however, the anti-inflammatory effect induced by EMD observed in the *in vitro* study could not be confirmed clinically.

Several studies have demonstrated that enamel matrix proteins, in particular amelogenin, applied to the root surface during periodontal surgery promote periodontal regeneration in pre-clinical^1^ and in clinical studies^2^. Clinical studies on enamel matrix proteins have focussed on healing periods varying between 6 months and 3 years^2^,^3^. Early healing events are critical and dictate the type of tissue that will develop later^1^,^4^,^5^. Histological studies^6^ have suggested that enamel matrix proteins are detectable on the denuded tooth root surface for 2–4 weeks following surgery. This seems to be a sufficiently long period of time to permit recolonization of periodontal ligament cells, initiate regenerative pathways and inhibit the down growth and proliferation of epithelial cells^4^. It is crucial to understand early healing mechanisms and use the knowledge gained to develop improved therapeutic strategies to promote regrowth of periodontal tissues lost due to disease. Moreover, clinical reports have suggested that enamel matrix proteins lead to improved early soft tissue wound healing^7^,^8^. Based on these clinical observations, it has been suggested that EMD may have an effect on gingival fibroblasts; relevant cells in soft tissue wound healing^9^. The gingival connective tissue is the oral equivalent to the dermis of the skin, and the main residing fibroblast is the gingival fibroblast^10^. Fibroblasts have a central role in wound healing as they do not only produce and organize the ECM^11^,^12^,^13^ but are also able to regulate inflammation through chemokine and cytokine expression^14^, angiogenesis^15^, and re-epithelialization^16^,^17^. EMD-induced cytokine release on gingival fibroblasts during the early healing period contribute to a possible favourable early wound healing effect^18^ or even play a role in the subsequent EMD-mediated regeneration of the tissues.

Several *in vitro* studies have provided valuable information on the molecular mediators induced by EMD^19^,^20^. In periodontal ligament (PDL) fibroblasts, EMD increased interleukin-6 (IL-6), TGF-β1 and PDGF-AB production^21^ and reduced IL-4 gene expression^22^. In an extensive analyses of cytokines secreted from PDL cells^23^, higher molecular weight fractions of EMD were found to induce an increase in vascular endothelial growth factor (VEGF) and IL-6 secretion, whereas lower molecular weight fractions enhanced cell proliferation and secretion of IL-8 and monocyte chemoattractant protein-1 (MCP-1) and reduced IL-4 release.

*In vitro* studies are often used as models to indicate and evaluate molecular mechanisms of clinical treatment strategies. GCF components have been studied with the aim of using them as predictors of periodontal disease progression and healing after therapy^24^, but with little success^24^. It is likely that a diagnostic model utilising a broader spectrum of GCF components would have a greater predictive power compared to models based on few components.

The aim of the present study was to assess and compare the effect of enamel matrix derivative (EMD) on the cytokine profiles from human gingival fibroblasts *in vitro* and, clinically, in gingival crevicular fluid during early periodontal wound healing. In addition, the bioactivity of various fractions of EMD was evaluated *in vitro* to identify active components.

## Results

### Clinical study

An overview of the cytokine profile induced by EMD and control groups is shown in Table 1. There were no adverse events or discomforts associated to the EMD or the control group. There were no significant differences between EMD- and control-treated sites at baseline for any of the cytokines measured.

**Figure 1:**
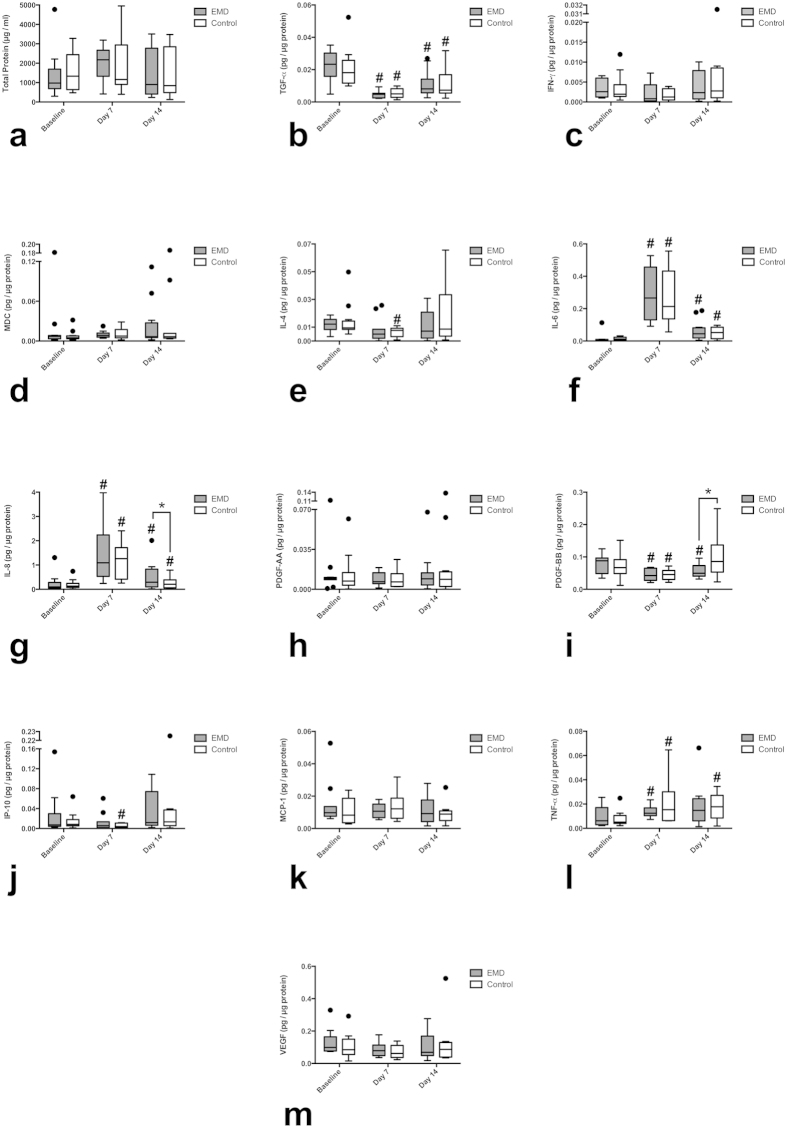
Box plot of GCF normalized cytokine levels (pg cytokine/μg total protein) at baseline, day 7 and day 14. (**a**) Total protein, (**b**) TGF-α, (**c**) IFN-γ, (**d**) MDC, (**e**) IL-4, (**f**) IL-6, (**g**) IL-8, (**h**) PDGF-AA, (**i**) PDGF-BB, (**j**) IP-10, (**k**) MCP-1, (**l**) TNF-α, (**m**) VEGF. ^#^Significant intra-group differences in cytokine levels between baseline and day 7 or day 14 (p < 0.05). *Significant inter-group differences in cytokine levels between control and EMD sites (p < 0.05). Generalized estimating equations (GEE) (n = 12 patients). Box plots show the median, the 25^th^ and 75^th^ percentiles, Tukey whiskers (median ± 1.5 times interquartile range), and outliers (•).

TGF-α was significantly lower in both control (p = 0.0001) and EMD (p = 0.0001) at day 7 compared to baseline TGF-α levels. At day 14, TGF-α values were also significantly reduced in both the control (p = 0.022) and EMD (p = 0.003) groups compared to baseline values. There were no significant differences between the groups at any time point studied (Fig. 1b).

IL-4 levels in GCF were reduced in both the test and control group at day 7 compared to baseline values, however, it was only significantly changed (p = 0.014) for the control group. There were no significant differences between the two groups at day 7 and day 14. (Fig. 1e).

IL-6 was enhanced 50-fold in EMD-treated sites (p < 0.0001) and 25 fold in the control group (p < 0.0001) compared to baseline values at day 7. The difference between groups did not reach statistical significance. At day 14, IL-6 was also significantly increased 12-fold and 5-fold in EMD (p = 0.009) and control groups (p < 0.0001) respectively, compared to baseline values. There were no significant differences between the groups at any time point (Fig. 1f).

Interestingly, EMD-treated sites presented significantly increased levels of IL-8, around 25-fold (p < 0.0001) at day 7 and 5-fold (p = 0.006) at day 14 compared to baseline values. The control group also presented significantly increased levels of IL-8, around 11-fold (p < 0.0001) at day 7 and 2-fold (p < 0.0001) at day 14 compared to baseline values. Significant inter-group differences were found at day 14 with EMD-treated sites having a more pronounced effect on IL-8 release than in the control group (p = 0.030) (Fig. 1g).

PDGF-BB levels in GCF were significantly decreased in EMD both at day 7 (p = 0.001) and day 14 (p = 0.021) compared to baseline levels. By contrast, PDGF-BB levels in control sites were significantly reduced at day 7 (p = 0.001) and were increased twice at day 14, although this increase was not significant. EMD-treated sites significantly decreased PDGF-BB levels at day 14 compared to the control sites (p = 0.042) (Fig. 1i).

TNF-α release was significant at day 7 in the EMD and control groups, with an increase of 3-fold (p = 0.044) and 4-fold (p = 0.021) respectively, compared to baseline values. The release of TNF-α was also significantly enhanced 3-fold in the control group (p < 0.0001) at day 14 compared to baseline values. There were no differences between groups at any time point (Fig. 1l).

IP-10 release was unchanged at day 7 in both groups. A significant intra-group difference was found at day 7 in the control group compared to baseline values (p = 0.013). There were no significant differences between groups at any time point (Fig. 1j).

There were no significant intra-group or inter-group differences for total protein, IFN-γ, MDC, PDGF-AA, MCP-1 and VEGF at any of the time points assessed.

### *In vitro* study

An overview of changes in cytokine levels for the different fractions is given in Table 2. In contrast to clinical findings, EMD reduced the IL-6, IL-8 and VEGF secretion compared to untreated gingival fibroblasts during the first week of the experiment.

Similarly, both high and low molecular weight fractions of EMD reduced IL-6, IL-8, VEGF and MCP-1 secretion compared to untreated cells over the 2-week study period. No differences between high and low molecular weight fractions were observed in terms of inducing cytokine secretion.

The anti-inflammatory cytokine profile obtained by the various fractions in the *in vitro* study could not be confirmed clinically.

While IL-4, IP-10, TNF-α and IFN-γ levels could be detected in GCF in the clinical study, these cytokines were below the detection limit for the assay and for all treatment groups at all time points in the *in vitro* study. Other cytokines that were below the detection limit in the *in vitro* study were: G-CSF, IL-7 and RANTES.

## Discussion

The observed effect of EMD on the various cytokines secreted from gingival fibroblasts *in vitro* was not replicated in GCF from treated periodontal patients. The discrepancy between the *in vitro* and clinical results may be related to contribution of factors from more than one cell type in GCF. In addition, the EMD used in the clinical study was from a different lot number than the EMD used *in vitro* and being a biologically derived product, the content of EMD might vary between different batches. Differences among lots of EMD will have to be determined.

TGF-α levels in GCF were reduced in both control and EMD treated sites compared to baseline values over the 2-week study period. TGF-α plays an important role in the proliferative phase of wound healing as it stimulates epidermal regrowth, angiogenesis and granulation tissue formation^25^,^26^,^27^. Nonetheless, TGF-α has been shown to be significantly reduced in patients with periodontal disease compared to healthy patients^28^. Altered TGF-α secretion in periodontitis patients may affect periodontal wound healing.

Among all cytokines analyzed, the pro-inflammatory cytokines IL-6 and IL-8 were the cytokines most influenced by periodontal surgery in both EMD and control sites. Levels of these two cytokines are elevated during the inflammatory phase of wound healing^29^,^30^. Engelhardt *et al*.^29^ demonstrated enhanced IL-8 expression in the superficial wound bed that correlated spatially and temporally with neutrophil infiltration. Galluci *et al*.^30^ identified IL-6 expression in the epithelial wound edge and in macrophages and fibroblasts of the dermis.

In the *in vitro* study, EMD and its fractions induced a reduced secretion of pro-inflammatory factors. This observation is in line with Nokhbehsaim *et al*.^31^ who found that EMD downregulated IL-1β, IL-6 and IL-8 expression in PDL cells. A reduction of IL-6, IL-8 and MCP-1 secretion during the early phase of healing might lead to a reduced migration of inflammatory cells at the wound site^32^,^33^,^34^. In contrast, EMD and its fractions have been found to enhance the secretion of IL-6, IL-8 and MCP-1 in PDL fibroblasts^23^. This discrepancy might be explained by the difference in characteristics and functions of these cells^35^. EMD contains hundreds of peptides in different levels of proteolytic processing that may provide a range of biological effects of importance in wound healing^23^. It is possible that the effect of EMD in wound healing and regeneration may be related to a single component or, on the other hand, its effect may be caused by its composition as a whole, or a range of molecular weight components. This question remains unanswered in the literature. There are difficulties to characterize EMD because, upon size fractionation, the same proteins may be found in several fractions^23^. There is the possibility that a purified bioactive component can be characterized and thus an improved EMD formulation may be developed.

Reports from clinicians have claimed that early wound healing is characterized by a reduced inflammation in the gingival soft tissue^7^,^8^. The observed increase in pro-inflammatory cytokines in GCF may indicate contribution from other sources than gingival fibroblasts.

The total GCF protein content was increased, however, this did not reach significance compared to baseline values. Total protein has been shown to increase with gingival inflammation^36^,^37^. Giannopoulou *et al*.^38^ showed that after non-surgical periodontal therapy, total protein significantly decreased. This was accompanied with a decrease in clinical signs of inflammation^38^. Thus, it is expected that the total protein would increase during the early inflammatory phase of wound healing.

Villa *et al*.^23^ found that most enamel matrix derived fractions induced a decreased secretion of IL-4 in PDL fibroblasts compared to untreated cells. In the present clinical study, IL-4 secretion was decreased in both EMD and control groups compared to baseline levels, but only reached significance in the control group.

PDGF-AA levels were increased in both the control and EMD groups over the study period, although none of these effects were significant neither in the intra-group nor in the inter-group analyses. By contrast, PDGF-BB levels were significantly reduced by EMD compared to baseline values. This pattern was not found for the control group. It has been suggested that PDGF-A chains might play a role in periodontal wound healing during the first days of healing and PDGF-B chains might become more relevant in the wound bed later, correlating with fibroblast induced collagen synthesis that takes place after week 1^39^.

Examples where no significant changes in cytokine levels between test and control groups were observed may be related to high variation between individuals^40^ and the relatively low number of patients. Another issue to take into account is that some of the inflammatory cytokines analyzed might be constitutively overproduced in periodontitis lesions^41^. Thus, high levels of these cytokines might already be present at baseline and also during wound healing. This might explain why some cytokines might not show differences over time or between groups. It is generally accepted that the intra- and inter-assay variation should be below 15%^42^. 3 cytokines presented an inter-assay variation above 15%: IP-10 (15.3%), PDGF-AA (16.7%) and IL-6 (18.3%). Thus, the results have to be interpreted cautiously.

This study had a split-mouth design. An important drawback of such a design is the possibility of a carry-across effect^43^. The results have to be interpreted carefully as this type of design can lead to reduced treatment differences, and estimates of the treatment effect from split-mouth and parallel designs may not be the same^43^.

The most preferred strategy for collecting GCF is by use of paper strips. In the present study paper points were used. To our knowledge there is no evidence to support the use of paper strips over paper points to collect GCF. In one study^44^, paper strips showed higher levels of cytokines and a higher recovery rate than paper points. Nonetheless, these factors also depend on paper composition^45^ and binding properties of different cytokines which may be influenced by factors such as hydrophobicity, charge and structure^44^.

A common problem in GCF analyses is the low volume collected, which precludes the analyses of multiple cytokines^46^,^47^. Modern multiplexing methods such as Luminex require low sample volumes, are highly sensitive, and allow the analyses of up to 100 analytes simultaneously in one sample, a great advantage compared to methods measuring individual analytes (e.g. ELISA). Identification of cytokine profiles may increase the predictability of regenerative procedures. Future studies should establish which cytokine profiles are associated with regenerative outcomes and which profiles are related to unfavourable outcomes.

To the best of our knowledge this is the first report that simultaneously assesses cytokine secretion from human gingival fibroblasts and gingival crevicular fluid after the administration of EMD. In this report we are suggesting some mechanisms underlying the possible early healing effect of EMD. The assessment of postoperative healing with an adequate healing index^3^ and a cytokine profile might predict regenerative outcomes.

## Conclusions

From the findings of the present study and previously published work^23^, EMD may promote improved early wound healing with reduced gingival fibroblasts induced inflammation. The regenerative capabilities of EMD might be mediated through its influence on PDL fibroblasts. The anti-inflammatory cytokine profile observed *in vitro* could not be confirmed clinically. In addition, EMD affects growth factors and cytokines related to fibroplasia, angiogenesis, inflammation and chemotaxis both *in vitro* and *in vivo.*

## Material and Methods

### Clinical study

The clinical study was designed as a randomised controlled split-mouth clinical trial. The study protocol was approved by the Regional Committee for Research Ethics, Oslo, Norway (2013/1827/REK sør-øst C), and patients signed an (approved) informed consent form before entering the study. The investigation was performed according to the principles of the Declaration of Helsinki on experiments involving human subjects. The presence of biological markers in the GCF was assessed at baseline, and one and two weeks postoperatively.

### Patients

Subjects undergoing elective periodontal treatment at a private periodontal specialist clinic were included in the present study. Fifteen patients aged 25–75 years suffering from generalized severe chronic periodontitis were recruited during the first trimester of 2014 and treated during the period between April and August of 2014. Three subjects that required grafting procedures were excluded from the study and one patient did not attend the surgery for the control site, leaving a total number of 11 sites for the control group and 12 sites for the EMD treated group. Adverse events were evaluated and recorded at each visit. All patients had undergone an initial causative treatment phase consisting of patient motivation, oral hygiene instruction and supra-, subgingival scaling and root planing. Patients were re-evaluated six weeks after scaling and root planning and scheduled for surgery as appropriate. Baseline measurements at the test and control sites were obtained on the day of surgery. Baseline defect characteristics are presented in Table 3.

### Inclusion criteria and exclusion criteria for participation in the study

The inclusion criteria were based on the presence of two similar periodontal defects in different quadrants with clinical attachment loss of at least 5 mm, probing pocket depth of 6 mm or more, horizontal and/or vertical bone loss as demonstrated by the probing measurement and radiographic assessments following the initial phase of periodontal treatment. Experimental teeth must either have exhibited a vital pulp or, if subjected to root canal treatment, be asymptomatic.

Exclusion criteria were smoking, patients having used antibiotics in the 6 months prior to treatment, patients with disorders that compromise wound healing, such as diabetes mellitus, cancer, HIV, chronic high dose steroid therapy, radiation or immune-suppressive therapy and patients with acute infectious lesions in the area of intended therapy.

### Surgical intervention

Clinical measurements were obtained at baseline. Full-thickness mucoperiosteal flaps were elevated on the buccal, lingual and interproximal aspects of the involved teeth. Granulation tissue adherent to the alveolar bone was removed. Remaining subgingival calculus was removed with curettes. Following open flap debridement, teeth were randomly assigned to receive either the test or the control treatment by the tossing of a coin. In the test sites, EMD in propylene glycol alginate (Emdogain; Straumann, Basel, Switzerland) was directly applied to the exposed alveolar bone as well as onto the root surfaces. Flaps were repositioned and tension-free primary closure of the wound was achieved by means of single non-resorbable sutures (Propylen 5.0; Medipac, Kilkis, Greece). Control sites received the same surgical procedure but without the application of Emdogain. Sutures were removed after 14 days. Patients were advised to rinse twice daily with a 0.2% solution of chlorhexidine digluconate (Corsodyl; GlaxoSmithKline, Münchenbuchsee, Germany) for 2 weeks. No antibiotics were given^48^,^49^, and mechanical oral hygiene was not allowed in the surgical areas during this period. Analgesics were taken by the patients on an as needed basis.

### Gingival crevicular fluid (GCF) sampling

GCF samples were obtained from both test and control sites at baseline, day 7 and day 14 post-surgery as previously described^50^. Multiplex analyses of the level of cytokines in GCF was performed as previously described^50^. The experimental sites were isolated with cotton rolls, and GCF was collected using endodontic paper points ISO size 50 (Beutel rock VDW; München, Germany) in the gingival sulcus for 10 seconds at the experimental sites of control and test teeth. The paper points were transferred to Eppendorf microtubes (Axygen, Tewksbury, MA, USA) and were coded with a unique identifier. Samples were immediately frozen at −20 °C after collection, and transferred and stored at minus 80 °C until analysis.

### Protein quantification from gingival crevicular fluid

All laboratory analyses were performed blind with respect to sample identification, visit and patient information. Tris-buffered saline (TBS; 200 μl) was added to each tube containing GCF samples. Multianalyte profiling of the level of cytokines was performed on the Luminex-200 system (Luminex, Austin, TX, USA). The levels were monitored related to the amount of total protein in each sample. The amount of TGF-α, interferon-γ (IFN-γ), human macrophage-derived chemokine (MDC/CCL22), PDGF-AA, PDGF-BB, IL-4, IL-6, IL-8, interferon-γ induced protein (IP-10), MCP-1, tumor necrosis factor-α (TNF-α), and VEGF, was measured using the human cytokine/chemokine kit (Milliplex human cytokine MPXHCYTO-60k, Millipore, Billerica, MA, USA). All analyses were performed according to the manufacturers’ protocols. The intra-assay and inter-assay variation for the cytokines analyzed ranged between 1.5 and 18.3%.

The total protein content in GCF was determined using the BCA protein assay kit (Pierce, Rockford, IL, USA) following the manufacturer’s kit instructions.

### *In vitro* study

#### Cell cultures

Commercially available human gingival fibroblasts (ATCC, Manassas, VA, USA) were grown in Dulbecco’s modified Eagle’s medium (High Glucose 4.5 g/l, PAA laboratories, Linz, Austria) supplemented with 10% fetal bovine serum and 100 U/ml penicillin and 100 μg/ml Streptomycin (PAA laboratories). Cells were subcultured at 37 °C in a humidified atmosphere of 10% CO_2_ according to the manufacturer’s instructions.

#### EMD fractions

Fractions were obtained as described previously^23^. Briefly, EMD (Biora, Malmö, Sweden) was subjected to size-exclusion chromatography using a 90 × 1.6-cm column of Bio Gel P10 (Bio-Rad, Hemel Hempstead, UK). Proteins were eluted using 0.125 M formic acid and the column eluent was monitored at 280 nm and 5-ml fractions were collected and freeze dried. The cultured cells were treated with either EMD or the various fractions reconstituted in cell culture medium at molar concentrations equivalent to their content in 10 μg ml^−1^ of whole EMD. Untreated cells were used as controls. Three replicate experiments were conducted for every fraction, EMD and the controls. Cell culture media was harvested after 1, 3, 7 and 14 days. The fractions were characterized by sodium dodecyl sulphate polyacrylamide gel electrophoresis (SDS-PAGE) and liquid chromatography electrospray ionization–tandem mass spectrometry as previously described^23^.

#### Protein quantification in cell culture medium

Multianalyte profiling of the level of cytokines in concentrated cell culture medium of gingival fibroblasts was performed on the Luminex-200 system (Luminex) as previously described^23^. The amount of granulocyte colony-stimulating factor (G-CSF), IL-4, IP-10, TNF-α, IFN-γ, IL-6, IL-7, IL-8, MCP-1, regulated upon activation of normal T-cell expressed and secreted (RANTES) and VEGF was measured using the human cytokine/chemokine kit (Milliplex human cytokine MPXHCYTO-60k; Millipore). The intra-assay variation for the analyzed cytokines varied between 1.5 and 4.3%. The inter-assay variation varied between 3.5% and 18.3%.

#### Statistical analysis

Sample size calculation was not performed, as this study was a pilot to assess the effect of EMD on multiple cytokines after periodontal surgical therapy. For the clinical study, cytokine concentrations were normalized to total protein (pg of cytokine μg total protein^−1^) and served as the primary outcome. Generalized estimating equations were used to adjust for clustering within subjects in a split-mouth design and to investigate the interaction between GCF cytokine levels (dependent variable) and time or treatment group (independent factors). Thus, inter-group comparisons in cytokine levels were made at each time point and intra-group comparisons in cytokine levels were made to baseline values at day 7 and day 14. This statistical analyses was performed with SPSS for Mac (version 21; SPSS, Chicago, IL, USA) (Supplementary Dataset).

For the *in vitro* study, statistical comparison between treatment groups and controls was performed using parametric one-way analysis of variance (ANOVA) and post-hoc Dunnet’s test with multiple comparisons towards the untreated control group. When the normality test failed, ANOVA on ranks (Kruskal Wallis test) and post-hoc Dunn’s test was performed. (SigmaStat software; Systat, San Jose, CA, USA). P ≤ 0.05 was considered significant.

## Additional Information

**How to cite this article**: Villa, O. *et al*. EMD in periodontal regenerative surgery modulates cytokine profiles: A randomised controlled clinical trial. *Sci. Rep.* **6**, 23060; doi: 10.1038/srep23060 (2016).

## Supplementary Material

Supplementary Information

## Figures and Tables

**Table 1:** Cytokine profile induced by EMD- and controlled-operated sites during early periodontal wound healing.

	Day 7	Range day 7	Day 14	Range day 14
EMD	↑Total protein	5227.14	↑Total protein	4362.86
	↓TGF-α^#^	0.03	↓TGF-α^#^	0.05
	↓IFN-γ	0.01	↑IFN-γ	0.01
	↑MDC	0.01	↑MDC	0.20
	↓IL-4	0.03	↑IL-4	0.03
	↑IL-6^#^	0.40	↑IL-6^#^	0.21
	↑IL-8^#^	3.80	↑IL-8^#^,*	1.18
	↑PDGF-AA	0.02	↑PDGF-AA	0.06
	↓PDGF-BB	0.12	↓PDGF-BB*	0.12
	↓IP-10	0.03	↑IP-10	0.14
	↑MCP-1	0.05	↑MCP-1	0.06
	↑TNF-α^#^	0.02	↑TNF-α	0.08
	↓VEGF	0.15	=VEGF	0.38
CONTROL	↑Total protein	6400.00	↑Total protein	5531.43
	↓TGF-α^#^	0.05	↓TGF-α^#^	0.06
	↓IFN-γ	0.01	↑IFN-γ	0.04
	↑MDC	0.02	↑MDC	0.21
	↓IL-4^#^	0.03	↑IL-4	0.10
	↑IL-6^#^	0.51	↑IL-6^#^	0.11
	↑IL-8^#^	2.12	↑IL-8^#^,*	0.94
	↑PDGF-AA	0.05	↑PDGF-AA	0.16
	↓PDGF-BB	0.10	↑PDGF-BB*	0.25
	↓IP-10^#^	0.04	↑IP-10	0.27
	↑MCP-1	0.03	↑MCP-1	0.03
	↑TNF-α^#^	0.08	↑TNF-α^#^	0.03
	↓VEGF	0.24	↑VEGF	0.71

Cytokines after symbol ↑, ↓ or = mean that they are increased, decreased or constant respectively based on mean values of fold changes compared to baseline cytokine levels. Total protein values are given in μg ml^−1^. Cytokine levels are given in pg cytokine μg total protein^−1^. The range is calculated from changes with respect to baseline levels. Only statistically significant changes marked with * or ^#^ define whether a cytokine is changed. ^**#**^Significant intra-group differences compared to baseline cytokine levels (p < 0.05). *Significant inter-group differences at day 7 or day 14 (p < 0.05) (n = 12 patients).

**Table 2:** Cytokine levels in cell culture medium from gingival fibroblasts incubated with EMD and EMD fractions (F1–F13) relative to untreated control at the same timepoint.

Gingival fibroblasts	Day 1	Day 3	Day 7	Day 14
EMD	↓IL6**	↓VEGF*	↓IL8**	
F1		↓IL8**, VEGF**	↓IL6***, IL8**	↓IL6**, IL8**, MCP1**, VEGF*
F2		↓IL6**, MCP1**, VEGF***	↓IL6**, IL8**, MCP1**	
F3	↓VEGF**	↓VEGF***	↓MCP1**	
F4		↓IL6***, IL8**, MCP1***, VEGF***		
F5	↓IL6**, IL8**, MCP1**, VEGF**	↓IL6**, VEGF**	↓IL6***, MCP1**	
F6	↓IL6**	↓IL6***, IL8**, VEGF***	↓IL6**	↓IL6***, IL8***, MCP1***, VEGF**
F7		↓IL6**, IL8***, MCP1**, VEGF***	↓IL8**, MCP1**	↓IL6**
F8		↓VEGF**		
F9			↓IL6***, IL8***, MCP1***	↓IL6**, IL8**, MCP1***, VEGF**
F10		↓VEGF*		
F11				
F12		↓VEGF*		
F13				

EMD: enamel matrix derivative. IL-6: interleukin-6; IL-8: interleukin-8; MCP-1: monocyte chemoattractant protein-1; VEGF: vascular endothelial growth factor. ↓ mean that cytokine levels are significantly reduced compared to the control group. Fractions 1–6 shared components above or around 20 KDa, whereas fractions 7–13 contained components below 5 kDa. *p < 0.05, **p < 0.01, ***p < 0.001 (n = 3).

**Table 3:** Baseline characteristics of the periodontal defects.

Variable	EMD (n = 12)	CONTROL (n = 11)	P value
PPD at deepest defect (mm)	7.0 (6, 8)	7.0 (6, 8)	n.s.
CAL at deepest defect (mm)	6.0 (5, 8)	6.0 (6, 7)	n.s.
Deepest infrabony defect (mm)	2.5 (0, 8)	2.0 (0, 5)	n.s.
Total protein (μg ml^−1^)	975.57 (4475.71)	1307 (2802.86)	n.s
TGF-α (pg ml^−1^)	28.04 (94.25)	22.3 (64.03)	n.s.
IFN-γ (pg ml^−1^)	2.47 (4.65)	3.2 (10.1)	n.s.
MDC (pg ml^−1^)	7.39 (116.75)	8.3 (14.08)	n.s.
IL-4 (pg ml^−1^)	12.04 (17.88)	15.16 (29.32)	n.s.
IL-6 (pg ml^−1^)	7.61 (50.66)	13.46 (37.29)	n.s.
IL-8 (pg ml^−1^)	96 (1581.8)	186 (422.67)	n.s.
PDGF-AA (pg ml^−1^)	9.6 (73.35)	11.84 (30.13)	n.s.
PDGF-BB (pg ml^−1^)	77.09 (189.93)	80.74 (186.79)	n.s.
IP-10 (pg ml^−1^)	7.63 (96.26)	11.7 (31.81)	n.s.
MCP-1 (pg ml^−1^)	12.64 (40.02)	11.37 (26.95)	n.s.
TNF-α (pg ml^−1^)	6.28 (39.09)	7.18 (22.48)	n.s.
VEGF (pg ml^−1^)	116.02 (819.13)	131.92 (193.36)	n.s.

Clinical data given as median (min, max). Cytokine baseline levels data given as median (range). Cytokine levels were normalized to total protein (pg cytokine μg total protein^−1^). PPD: probing pocket depth. CAL: clinical attachment level. Comparisons between EMD and control treated sites. Clinical parameters analysed by Mann-Whitney U test. Cytokine parameters analysed by GEE; n.s. (non- significant).
